# The Incidence and Causes of Unplanned Reoperations as a Quality Indicator in Pediatric Surgery

**DOI:** 10.3390/children9010106

**Published:** 2022-01-13

**Authors:** Miro Jukić, Ivona Biuk, Zenon Pogorelić

**Affiliations:** 1 Department of Pediatric Surgery, University Hospital of Split, Spinčićeva 1, 21000 Split, Croatia; zpogorelic@gmail.com; 2 Department of Surgery, School of Medicine, University of Split, Šoltanska 2, 21000 Split, Croatia; biukivona@gmail.com

**Keywords:** unplanned reoperation, uROR, pediatric surgery, quality indicator, quality of surgical care, complications, treatment outcome

## Abstract

Background: Unplanned return to the operating room (uROR) within the 30-day postoperative period can be used as a quality indicator in pediatric surgery. The aim of this study was to investigate and evaluate uROR as a quality indicator. Methods: The case records of pediatric patients who underwent reoperation within the 30-day period after primary surgery, from 1 January 2018 to 31 December 2020 were retrospectively reviewed. The primary outcome of the study was the rate of uROR as a quality indicator in pediatric surgery. Secondary outcomes were indications for primary and secondary surgery, types and management of complications, factors that led to uROR, length of hospital stay, duration of surgery and anesthesia, and starting time of surgery. Results: A total of 3982 surgical procedures, under general anesthesia, were performed during the three-year study period (2018, *n* = 1432; 2019, *n* = 1435; 2020, *n* = 1115). Elective and emergency surgeries were performed in 3032 (76.1%) and 950 (23.9%) patients, respectively. During the study period 19 (0.5%) pediatric patients, with the median age of 11 years (IQR 3, 16), underwent uROR within the 30-day postoperative period. The uROR incidence was 6 (0.4%), 6 (0.4%), and 7 (0.6%) for years 2018, 2019, and 2020, respectively (*p* = 0.697). The incidence of uROR was significantly higher in males (*n* = 14; 73.7%) than in females (*n* = 5; 26.3%) (*p* = 0.002). The share of unplanned reoperations in studied period was 4.5 times higher in primarily emergency surgeries compared to primarily elective surgeries (*p* < 0.001). The difference in incidence was 0.9% (95% CI, 0.4–1.4). Out of children that underwent uROR within the 30-day period after elective procedures, 50% had American Society of Anesthesiologists (ASA) score three or higher (*p =* 0.016). The most common procedure which led to uROR was appendectomy (*n* = 5, 26.3%) while the errors in surgical technique were the most common cause for uROR (*n* = 11, 57.9%). Conclusion: Unplanned reoperations within the 30-day period after the initial surgical procedure can be a good quality indicator in pediatric surgery. Risk factors associated with uROR are emergency surgery, male gender, and ASA score ≥3 in elective pediatric surgery.

## 1. Introduction

Complications in surgery are a public health issue as their consequences can be financial, social, legal, and professional. The reported incidence of 30-day postoperative complications in patients who underwent general surgical procedures ranges from 5.8% to 43.5% [1]. Consequently, there is increased attention regarding the large number of hospitalizations for postoperative complications. It is believed that many of those complications can be avoided, including unplanned reoperations [2]. Therefore, there is an increasing demand to define measures of outcomes to improve surgical quality. Most used quality indicators in pediatric surgery are mortality, morbidity, unplanned readmissions, and the incidence of postoperative complications [3,4]. Many of those, such as mortality and morbidity, are often not applicable to the pediatric population because the incidence of those events is too low in children [5,6,7,8].

Unplanned reoperation rate could be a useful quality indicator in surgery, and even more in pediatric surgery. It is a broadly applicable outcome measure because unplanned reoperations are not specific, meaning they can occur after any surgical procedure [9,10]. Unplanned reoperations are also reliable because they are only performed when necessary and their incidence is easily tracked using administrative registries [11,12]. The incidence of unplanned reoperations in pediatric surgery varies from 0.8 to 7% in general pediatric surgery and up to 17% in pediatric neurosurgery [13,14]. Information about unplanned reoperations can be used to compare different hospitals, different wards, and even to make inside evaluation of surgical teams and surgeons. That way, one can find an adequate health care facility for the required procedure. Furthermore, evaluating and tracking postoperative complications such as unplanned reoperations can raise awareness of complications and surgical errors [13]. Identifying risk factors for unplanned reoperations could help to improve quality of surgery, minimize duration of hospital stay and the increased cost of treatment when unplanned reoperation occurs.

The aim of the study was to determine rate of unplanned reoperations or an unplanned return to operating room (uROR) in the 30-day postoperative period. An unplanned reoperation is defined as an operation that results as a complication of the primary procedure.

## 2. Materials and Methods

### 2.1. Patients

A retrospective analysis of all surgical procedures under general anesthesia during the three-year period in the Clinic for Pediatric Surgery at the University Hospital of Split was performed. Patient data was collected from registers and archives from 1 January 2018 to 31 December 2020. The study includes all patients who underwent elective or emergency surgery under general anesthesia within the investigated study period and evaluates unplanned reoperations and patient data. The patients of both genders, aged 0–18 years, who underwent either elective or urgent procedure in the investigated study period with an unplanned reoperation in the 30-day postoperative period were included in the study. Exclusion criteria were all patients older than 18 years of age, patients reoperated out of the previously defined study period, those who had an unplanned reoperation after the 30 days postoperatively or patients who had a planned reoperation in the 30-day period, and not as a complication of the primary procedure, patients who had a surgery as a part of the ‘one day surgery’ program or an outpatient clinic.

### 2.2. Outcomes of the Study

Primary outcome of the study was to determine the rate of uROR as a quality indicator. Secondary outcomes were indications for primary and secondary surgery, types and management of complications, factors that led to uROR, length of hospital stay, duration of surgery and anesthesia, and starting time of surgery.

### 2.3. Data Collection and Study Design

All the patient data was retrospectively collected from administrative registries and archives. For each patient the following variables were recorded: age, gender, body mass index (BMI), comorbidities, indication for the primary and unplanned reoperation, type of the first surgery (elective/emergency), length of hospital stay after the first and second (unplanned) operation, time in between surgeries, starting operating time, duration of the operation and anesthesia, the American Society of Anesthesiologists (ASA) classification, and the National Nosocomial Infections Surveillance (NNIS) index. The causes for the unplanned reoperation were classified as previously in the literature: errors in surgical technique, errors in management, patient’s other illness, and other causes outside the surgical department. For each patient who had an unplanned reoperation an extensive medical documentation was analyzed, including primary diagnosis that led to surgery, the course and duration of the primary operation, anesthesiologists record, everyday nurses reports and any other notable events during hospitalization. After thorough analysis, all senior pediatric surgeons along with authors reached a consensus on the classifications for the reasons of unplanned reoperation.

In more details, we defined an error in surgical technique as a result of not following the accepted protocol for routine operation and properly executing the cutting, reconstruction, and suturing. Pediatric surgeons agree that these types of mistakes and errors mostly occur under the pressure of timeframes, hour of the surgery, or some other external circumstances.

Errors in management are, therefore, defined a mistake in judgment and choice of treatment type. Additionally, all mistakes in management of wound dressing, management of drains, errors in transportation, and other would be prescribed to this category.

Patients were additionally screened for surgical complications according to Clavien-Dindo classification [15].

The study was approved by the Ethics Review Board of University Hospital of Split (reference No. 500-03/20-01/09; date of approval: 30 October 2020).

### 2.4. Statistical Analysis

Statistical analysis was performed using the SPSS 19.0 program (IBM Corp, Armonk, NY, USA) and Microsoft Excel for Windows version 16.0 (Microsoft Corporation, Redmond, WA, USA). The Kolmogorov–Smirnov test was used to test whether the data comes from a normal distribution. Quantitative data was described using mean and standard deviation or by median and interquartile range. Categorical variables were described using absolute numbers and percentages. Difference between mean values for quantitative data was tested using one or two tailed Student’s t-test for independent variables, Mann–Whitney U-test (Wilcoxon rank sum). Categorical variables were tested using the Chi square test or its non-parametric alternative Fischer exact test. A two-sided *p* value < 0.05 was considered statistically significant.

## 3. Results

A total of 3982 operations under general anesthesia were identified in the three-year study period. Out of that number of surgeries in total, there were 1432 (36%), 1435 (36%), and 1115 (28%) surgeries in years of 2018, 2019, and 2020, respectively. The total number of unplanned reoperations in the 30-day postoperative period was 19 out of 3982 (0.5%). In regard to each individual year, the number of unplanned reoperations was 6/1432 (0.4%) in 2018, 6/1435 (0.4%) in 2019, and 7/1115 (0.6%) in 2020. There was no statistically significant difference in the rate of unplanned reoperations comparing the individual years (*p* = 0.697).

Out of 19 patients who had underwent an unplanned reoperation in the 30-day postoperative period, significantly higher male predominance was found; there were 5 (26.3%) females and 14 (73.7%) males (*p* = 0.002). Median age for all the pediatric patients was 11 years (IQR 3, 16), meaning that 75% (*n* = 14) of patients were older than 3 years of age. Median age for females was 16 years (IQR 7.5, 16.5), while the median age for males was 9.5 years (IQR 3, 13) (*p* = 0.213).

From 3982 surgical procedures in the investigated study period, 3963 patients had only one surgery, while 19 needed an unplanned reoperation. In the first population there were 2948 (74.4%) males compared to the 14 (73.7%) males who had an unplanned reoperation (*p* = 0.944). Number of females in the first group was 1015 (25.6%), and 5 (26.3%) in the second group (*p* = 0.944). Median age was 11 years in both groups (*p* = 0.872) (Table 1).

The number of emergency/elective surgeries, number of unplanned reoperations in the 30-day postoperative period, and their share in the total number of surgeries performed in the three-year study period is showed in Table 1.

The share of unplanned reoperations in studied period was 4.5 times higher in primarily emergency surgeries compared to primarily elective surgeries (*p* <0.001). The difference in incidence was 0.9% (95% CI, 0.4–1.4).

In the structure of 19 patients who underwent an unplanned reoperation, 11 (57.9%) primarily had an urgent operation, while 8 (42.1%) had an elective surgery.

Distribution of patients compared to the type of primary surgery in relation to the year in which surgery was performed showed no statistical significance (*p* = 0.611) (Table 2).

Table 3 shows demographic and clinical characteristics of patients who had an unplanned reoperation within the 30-day postoperative period.

In the study group, there were 10 patients who had at least one comorbidity, four (36.4%) of those were in the emergency and 6 (75%) in the elective group (*p* = 0.112).

The mean starting operating time, in 24 h time period, was 17.50 ± 4.5 h in the emergency group, while the elective group had the starting time at 9.20 ± 0.2 h (*p =* 0.003).

The median length of hospital stay for patients in emergency group was 14.7 ± 5.3 days and 11.25 ± 6 days in the group of patients who had an elective primary surgery (*p* = 0.201).

Time between the first surgery and unplanned reoperation in the 30-day postoperative period was 9.2 ± 6.1 days in the group of patients who underwent an elective primary surgery compared to 4.5 ± 2.2 days in the emergency group. The time between the surgeries was on average twice as long in the emergency group (95% CI, 0.46–9.1) (*p =* 0.051).

According to the ASA classification, in the emergency group 10 (90.9%) children had ASA score 1 and one patient had ASA score 3 (9.1%). In the group of patients who underwent an elective primary surgery, three (37.5%) patients had ASA score 1, one patient (12.5%) had ASA score 2, two (25%) had ASA score 3, and two (25%) had ASA score 4 (25%). None of the patients had ASA score 5 (*p* = 0.016).

In regard to the NNIS index, three (27.3%) patients in the emergency group and four (54.5%) patients in the elective group had NNIS index zero. NNIS index 1 was found in six (54.5%) patients who had an emergency surgery and two (25%) patients who underwent an elective procedure. Additionally, two (18.2%) patients in the emergency group had NNIS index 2, which was found in one (12.5%) patient from the elective group. Only one patient had NNIS index 3, and it was in the elective group (12.5%) (*p* = 0.659).

Table 4 shows ASA and NNIS classifications for patients who had an unplanned reoperation based on their primary operation (emergency/elective).

The diagnosis that accounted for the largest proportion of the unplanned reoperations in the 30-day postoperative period was acute appendicitis, which was always performed in an *n* emergency service (*n* = 5; 26.3%). All causes of uROR are listed in Table 5.

The most common indications for unplanned reoperations were intraabdominal abscess (*n* = 4, 21.1%), wound dehiscence (*n* = 3, 15.8%), and postoperative ileus (*n* = 3, 15.8%). Other indications were wound hematoma (*n* = 2, 10.5%), postoperative bleeding (*n* = 1, 5.3%), skin infection (*n* = 1, 5.3%), and skin necrosis (*n* = 1, 5.3%) (Table 6).

Surgical errors were found as the most common cause for the uROR (*n* = 11; 57.9%). Errors in management caused five unplanned reoperations (26.3%), while in two patients (10.5%) the cause of the reoperation was determined to originate outside the surgical department (Table 7).

According to Clavien–Dindo classification [15] and due to nature of our study, no complications were defined as grade I or II. One complication (*n* = 1, 5.3%) was defined as grade IIIa. Almost all of the complications (*n* = 17, 89.4%) were graded as IIIb. A single patient (*n* = 1, 5.3%) had a complication that was classified as grade IVa.

## 4. Discussion

Given that unplanned reoperations in the 30-day postoperative period are most often complications of the primary procedure, their incidence is commonly accepted as a measure of quality in surgery. In this study, we defined postoperative complications as any undesirable deviations from the usual postoperative course that are consequences of the primary procedure. By measuring the rate of unplanned reoperations and by identifying the risk factors, there can be improvements in the surgical techniques. In this study, the incidence and characteristics of unplanned reoperations as an indicator of surgical quality were examined. The unplanned return to the operating room occurred in 0.5% of all patients. Incidence of reoperations in literature ranges from 0.8% to 17%, depending on the surgery field [9,13,16,17,18,19]. By comparing the data, we can observe that incidence of unplanned reoperations in present study is very low, which would attribute to the great surgery quality in our department.

In this study, the number of male patients (73.7%) was statistically significantly higher than the number of female patients (26.3%). Those results are compatible with results in other similar studies [13,17,18,19]. It is important, though, to compare the study subjects to the overall population of pediatric patients operated on during the study period. By comparing those numbers, we can observe that both patients who had unplanned reoperations and those who only had one operation have the same male to female ratio. The same goes to the median of age—11 years is the median both in the group of patients who had an unplanned reoperation and in those who did not. Mukerji et al. reported the median age of 8 years, which is similar to the data in our study [17].

Among the patients who had an unplanned reoperation in the 30-day postoperative period, 52.9% of them underwent an emergency surgery, which has no statistical significance. However, if we look at the share of emergency operations in the total number of operations performed in the three-year study period, we can notice that the share of unplanned reoperations is four times higher in emergency surgery (1.2%) than in elective surgery (0.3%). Considering the above-mentioned fact, the conclusion that emergency surgery is a risk factor for unplanned reoperations in the postoperative period may be drawn. Guevara et al. in their study also found that incidence of reoperations in emergency surgery was two times higher than in elective surgery [19]. Emergency surgery was also recognized as a risk factor in other published studies [17,18]. A significant difference has been also seen when comparing starting times of surgeries. Early time of starting in elective surgery is mostly explained by the fact that elective programs run from 8 a.m. to 3.30 p.m. The same does not follow emergency surgeries—they can be performed any time in the day or night, 24/7. Still, mean time of starting surgery was 5.50 p.m. in emergency surgery. Possible reasons for that can be fatigue, lack of concentration and organization of doctors and other medical staff in the later hours. Additionally, during regular working hours in the hospital, almost all employed specialists are available, who are more versed for certain procedures, while only the surgeon on duty works in the emergency service. Despite the statistical significance, we could not declare later starting time as a risk factor because we lacked information for all the reoperated patients. Roy et al. showed that performing surgeries after 5 p.m. is a risk factor for unplanned reoperations [20]. Similarly, Li et al. stated that unplanned reoperations occur more often if the surgeries are finished after hours [13].

This study showed no statistical difference when comparing length of hospital stay based on primary surgery. Length of hospital stay in the emergency group was 14.7 days, and 11 in the elective group. Results differ slightly from those in the study made by other authors. Li et al. reported mean hospital stay of 21 days, while Guevara reported mean hospital stay of 19 days [13,19]. When observing time between the two operations, significant differences were found. In the group of patients who had an elective surgery, time between procedures was 9 days compared to patients who had an emergency surgery, in which that period was half as long—4.5 days. As a possible reason for such a longer time frame, we can state the fact that the most common indication for reoperation in emergency group was intraabdominal abscess, which often takes a longer time to manifest.

Although the study conducted by Li et al. showed that a higher NNIS index is related to a greater risk for reoperations, our study did not confirm that result [13]. Out of 19 patients who had an unplanned reoperation, only four of them had an NNIS index higher than one. There was also no difference when comparing types of primary surgery (elective/emergency).

Besides NNIS index, ASA classification is also often mentioned as a good risk indicator. In fact, studies by Guevara et al. and Jubbal et al. showed that ASA score higher than three is a risk factor for unplanned reoperations [18,19]. In our study, 26.3% of patients had ASA ≥ 3. However, a statistically significant difference was found between the two groups, based on the primary surgery. In the group of patients who had an emergency operation only 9% had ASA ≥ 3, compared to 50% of patients in the elective group. Based on that statistical significance, we concluded that ASA ≥ 3 is a risk factor for unplanned reoperations in the 30 days postoperative period for patients that had an elective primary procedure.

Most of the unplanned reoperations were related to abdominal pediatric surgery, and the most frequent was an appendectomy (26.3%), which was always an emergency operation. The same results were shown in a study by Li et al. [13]. Most frequently, the indications for reoperations were related to wound complications, which coincides with results in other studies [9,12,13]. Wound dehiscence, hematoma, infection, or necrosis of the skin accounted for more than a third of the reoperations. The second most common indication was intraabdominal abscess, the same as in the study by Li et al. Furthermore, this study suggests that errors in surgical technique (57.9%) were the most common cause for reoperation. Our results are comparable with the results in the study made by Birkmayer et al., but lower than in study of Kroon et al. [9,12].

Although the surgical quality in the clinic is satisfactory according to the rate of unplanned reoperations, there are some limitations of the present study. Namely, the study was undertaken retrospectively, based on archives and preexisting medical documentation which can always be faulty. Research was conducted in a three-year period on 3982 operated children, which is not a big sample for these kinds of conclusions. Moreover, neurosurgical and cardiosurgical patients were not included, because they had separate departments in our institution. Furthermore, we can point out the fact that the number of operations in 2020 was 22% lower than in previous years of the study. The most probable cause for that deviation can be the reorganization of the healthcare system that occurred because of the SARS-CoV-2 pandemic [21,22]. That had a great influence on lowering the number of hospitalizations and operations. Moreover, the percentage of unplanned reoperations in the postoperative period was twice as large as in both 2018 and 2019. A possible explanation can be that, during the pandemic, the number of elective procedures was decreasing so there would not be too many hospitalizations in a situation where there was not enough medical staff present.

Unplanned reoperations in the 30-day postoperative period are a very attractive and simple indicator of quality of surgery. This parameter can be used to compare hospitals or clinics, to increase transparency and to ensure that patients can choose the adequate healthcare. Moreover, this parameter is useful for doctors, as well. They can evaluate their work and educate themselves further so they can keep improving their work. To the future generations of surgeons, unplanned reoperations can be used as guidance to improve quality in hospitals. In addition, they can help to implement new methods of surgical work, as their success can be tracked by the rates of postoperative reoperations.

Recently, the same group of authors investigated 30-day readmission as an indicator of quality care in pediatric surgery [4]. The study was preformed analyzing patients from the same clinic. Results showed a 0.8% incidence of 30-days readmission, which is comparable to results in this study. Many of other results were similar: both studies identified emergency primary surgery as a risk factor. The most common procedure that led to both readmissions and uROR was appendectomy, surgical wound infections were leading indications for both and there were significantly more male than female patients in both studies. Using the incidence of uROR could be a better quality indicator in surgery as it is more field specific and offers more insight and valuable information for improvements in surgical quality.

Still, there are some factors that need to be controlled so that reoperations can be a good quality indicator. Data and registries from which information are taken should be well defined and objective. Studies made in the past showed that only prospective tracking of complications can lead to correct acquisition [9,23]. When using reoperations as a quality indicator, preoperative characteristics of a patient and the complexity of surgery performed should be also taken in consideration. In that way, reoperations can be a valid outcome measure. A study done by Shepers et al. showed that the rate of unplanned reoperations in bigger hospital centers was higher than in small hospitals [24]. That is one of the reasons why a faulty impression may happen, and it is very important to consider all factors. In evaluation of a surgeon’s work based on his rate of unplanned reoperations, there are potential problems that should be emphasized. By using reoperations as indicators of quality, surgeons can be discouraged to perform harder and more complex surgeries. That way, they could protect themselves from increasing reoperations rates and could seemingly save their image. Furthermore, it is possible that they do not report all unplanned reoperations or that they reject patients who have complicated medical diagnoses [9]. All of the above can be very harmful for patients.

## 5. Conclusions

The rates of unplanned reoperations in the 30-day postoperative period are a useful and reproducible indicator of quality in surgery. Risk factors for unplanned reoperations are emergency primary operation, male gender, and ASA ≥ 3 in patients with elective surgery. The most common procedure associated with reoperations is appendectomy. Most often the cause is an error in a surgical technique. Still, before using reoperations as a standard for quality in surgery, a few needs must be met. There should be a standardized, objective, and prospective registry of all unplanned reoperations. Additionally, it should be correlated with preoperative characteristics of patients and the complexity of the procedure itself.

## Figures and Tables

**Table 1:** Relation between number of emergency/elective surgeries in relation to the number of unplanned reoperations in the 30-day postoperative period and their share in the total number of surgeries performed in each individual year.

	Number of Surgeries		Number of Unplanned Reoperations (*n*)		Share of Unplanned Reoperations (%)		
Year	Emergency	Elective	Emergency	Elective	Emergency	Elective	*p* *
2018	353	1079	4	2	1.1	0.2	0.036
2019	322	1113	4	2	1.2	0.2	0.026
2020	275	840	3	4	1.1	0.5	0.373
Total	950	3032	11	8	1.2	0.3	<0.001

* Fisher exact test.

**Table 2:** Distribution of patients based on the type of primary surgery (elective/emergency) for each individual year.

	Year			p *
Type of Surgery	2018. (*n* = 6)	2019. (*n* = 6)	2020. (*n* = 7)	
Emergency (*n* = 11)	4 (66.7)	4 (66.7)	3 (42.9)	0.611
Elective (*n* = 9)	2 (33.3)	2 (33.3)	4 (57.1)	
Total (*n* = 19)	6	6	7	

* Fisher exact test.

**Table 3:** Demographic and clinical characteristics of patients who had an unplanned reoperation in the 30-day postoperative period.

	Emergency				Elective				*p*
	2018	2019	2020	Total	2018	2019	2020	Total	
	(*n* = 4)	(*n* = 4)	(*n* = 3)	(*n* = 11)	(*n* = 2)	(*n* = 2)	(*n* = 4)	(*n* = 8)	
Number of unplanned reoperations, *n* (%)	4/353 (1.1)	4/322 (1.2)	3/275 (1.1)	11/950 (1.2)	2/1079 (0.2)	2/1113 (0.2)	4/840 (0.5)	8/3032 (0.3)	<0.001 *
Male gender, *n* (%)	2 (50)	2 (50)	3 (100)	7 (63.6)	1 (50)	2 (100)	4 (100)	7 (87.5)	0.245 ^†^
Age, median (IQR)	13.5 (7, 16)	13 (3, 16)	13 (2, 15)	13 (6, 16)	1.5 (0.02, 3)	10.5 (4, 17)	9.5 (2, 16)	6.5 (1, 15)	0.261 ^‡^
Comorbidities, *n* (%)	1 (25)	1 (25)	2 (66.6)	4 (36.4)	2 (100)	1 (50)	3 (75)	6 (75)	0.112 ^†^
Body mass index (kg/m^2^)	18.18 ± 2.24	17.8 ± 1.6	21.97 ± 4	19.05 ± 3.2	15.8 ± 0.3	16.1 ± 1.1	17.83 ± 3.4	16.1 ± 2.1	0.165 ^‡^
Anesthesia duration, min	82.5 ± 54.4	77.5 ± 30	140 ± 45.8	96.5 ± 48.6	120 ± 70	65 ± 7	97.5 ± 45	95 ± 45	0.951 ^‡^
Operation duration, min	55 ± 45	48.75 ± 27.9	80 ± 42	57.5 ± 35.8	75 ± 35	35 ± 7	62.5 ± 35	58.75 ± 28.5	0.667 ^‡^
Operation starting time, h	22.40 ± 1.30	14.50 ± 2	17.25 ± 6	17.50 ± 4.5	9	0	9.40	9.20 ± 0.20	0.003 ^‡^
Hospital stay duration	15 ± 3.6	11.5 ± 6.6	18.7 ± 3	14.7 ± 5.3	10 ± 1.4	6 ± 1.4	14.5 ± 7.1	11.25 ± 6	0.201 ^‡^
Time between 1st and 2nd operation, days	8.75 ± 3.4	11.5 ± 10	7 ± 1	9.2 ± 6.1	6 ± 2.8	3.52 ± 1	4.25 ± 2.2	4.5 ± 2.2	0.051 ^‡^

IQR—interquartile range; * Chi square test; † Fisher exact test; ‡ two-tailed Student’s *t*-test.

**Table 4:** ASA and NNIS score in the study group based on the type of primary procedure (elective/emergency).

	Emergency (*n* = 11)	Elective (*n* = 8)	*p* *
ASA index, *n* (%)			0.016
ASA1	10 (90.9)	3 (37.5)	
ASA2	0	1 (12.5)	
ASA3	1 (9.1)	2 (25)	
ASA4	0	2 (25)	
ASA5	0	0	
NNIS index, *n* (%)			0.659
NNIS0	3 (27.3)	4 (50)	
NNIS1	6 (54.5)	2 (25)	
NNIS2	2 (18.2)	1 (12.5)	
NNIS3	0	1 (12.5)	

* Mann–Whitney U-test.

**Table 5:** Diagnoses that caused uROR in the 30-day postoperative period.

Total Number of uROR (*n* = 19)	Primary Diagnosis	*n*	%
PEDIATRIC ABDOMINAL SURGERY	Appendicitis	5	26.3%
*n* = 8 (42.1%)	Sacrococcygeal teratoma	1	5.3%
	Gastric lymphoma	1	5.3%
	Omphalocele	1	5.3%
PEDIATRIC UROLOGY/GINECOLOGY	Peritoneal catheter	2	10.5%
*n* = 6 (31.5%)	Hydrocele	2	10.5%
	Ureterolithiasis	1	5.3%
	Testicular torsion	1	5.3%
PEDIATRIC THORACIC SURGERY	Diaphragmatic hernia	1	5.3%
*n* = 1 (5.3%)			
PEDIATRIC GENERAL SURGERY	Cellulitis	1	5.3%
*n* = 4 (21%)	Abscess	1	5.3%
	Animal bite	1	5.3%
	Synovial sarcoma of the knee	1	5.3%

**Table 6:** Indications for unplanned reoperations.

Indication for Unplanned Reoperation	*n* (%)
Intraabdominal abscess	4 (21.1)
Wound dehiscence	3 (15.8)
Postoperative ileus	3 (15.8)
Wound hematoma	2 (10.5)
Wound infection	1 (5.3)
Skin necrosis	1 (5.3)
Postoperative bleeding	1 (5.3)
Other	4 (21)
Total	19 (100)

**Table 7:** Causes for uROR in the 30-day postoperative period.

Causes for Unplanned Reoperations	*n* (%)
Errors in surgical technique	11 (57.9)
Errors in management	5 (26.3)
Patient’s illness	2 (10.5)
Other	1 (5.3)
Total	19 (100)

## Data Availability

The data presented in this study is available upon request of the respective author. Due to the protection of personal data, the data is not publicly available.
